# Fuzzy Control Modeling to Optimize the Hardness and Geometry of Laser Cladded Fe-Based MG Single Track on Stainless Steel Substrate Prepared at Different Surface Roughness

**DOI:** 10.3390/mi13122191

**Published:** 2022-12-10

**Authors:** Maha M. A. Lashin, Mahmoud Z. Ibrahim, Muhammad Ijaz Khan, Kamel Guedri, Kuldeep K. Saxena, Sayed M. Eldin

**Affiliations:** 1 College of Engineering, Princess Nourah Bint Abdulrahaman University, Riyadh 11564, Saudi Arabia; 2 Department of Design and Production Engineering, Faculty of Engineering, Ain Shams University, Cairo 11517, Egypt; 3 Department of Mathematics and Statistics, Riphah International University, I-14, Islamabad 44000, Pakistan; 4 Department of Mechanical Engineering, Lebanese American University, Beirut P.O. Box 36, Lebanon; 5 Mechanical Engineering Department, College of Engineering and Islamic Architecture, Umm Al-Qura University, Makkah 21955, Saudi Arabia; 6 Department of Mechanical Engineering, GLA University, Mathura 281406, India; 7 Center of Research, Faculty of Engineering, Future University in Egypt, New Cairo 11835, Egypt

**Keywords:** laser cladding, geometry of track, surface hardness, fuzzy logic control system

## Abstract

Metallic glass (MG) is a promising coating material developed to enhance the surface hardness of metallic substrates, with laser cladding having become popular to develop such coatings. MGs properties are affected by the laser cladding variables (laser power, scanning speed, spot size). Meanwhile, the substrate surface roughness significantly affects the geometry and hardness of the laser-cladded MG. In this research, Fe-based MG was laser-cladded on substrates with different surface roughness. For this purpose, the surfaces of the substrate were prepared for cladding using two methods: sandpaper polishing (SP) and sandblasting (SB), with two levels of grit size used for each method (SP150, SP240, SB40, SB100). The experiment showed that substrate surface roughness affected the geometry and hardness of laser-cladded Fe-based MG. To predict and optimize the geometry and hardness of laser-cladded Fe-based MG single tracks at different substrate surface roughness, a fuzzy logic control system (FLCS) was developed. The FLCS results indicate that it is an efficient tool to select the proper preparation technique of the substrate surface for higher clad hardness and maximum geometry to minimize the number of cladding tracks for full surface cladding.

## 1. Introduction

Laser cladding is a promising coating method that uses laser power to melt the injected or preplaced coating material powder onto a substrate [1,2]. This technique is employed to enhance the surface properties of the substrate such as hardness, wear resistance, corrosion resistance, etc. [3,4,5]. Laser cladding leads to high adhesion strength due to the metallurgical bond developed between the coating layer and the substrate [2,6]. Additionally, laser cladding is a flexible process and can be easily controlled. Recently, extensive research has focused on developing metallic glass (MG) coating layers on metallic substrates because of their superior surface properties such as hardness, wear resistance, and corrosion resistance [7,8,9]. The commonly used coating technique is laser cladding due to its abovementioned features [10,11,12]. However, the amorphous structure of MGs is very sensitive to the laser cladding parameters such as laser power, scanning speed, laser beam spot size, injected powder flow rate, etc.; thus, selecting these parameters is a vital issue [13]. These parameters affect the properties as well as the quality and geometry of the cladded layer [14,15,16]. However, the substrate surface roughness has a significant effect on the geometry and hardness of the MG coating layer applied by laser cladding as reported previously [17]. To achieve higher MG coating hardness and better geometry, a reliable systematic optimization technique is thus required.

Soft computing and computational intelligence techniques are useful when exact mathematical information is not available, with these differing from conventional computing owing to their tolerance of imprecision, uncertainty, partial truth, approximation, and their metaheuristic nature. Many researchers have attempted to control the process parameters to optimize or predict the quality and geometry of the cladded layer either experimentally [18,19] or numerically [20,21]. A fuzzy logic system is one part of computational intelligence that depends on numerical data supplied by manufacturers. In some cases, the information and data about the process are limited due to process cost or complexity. A fuzzy logic system uses knowledge tidbits, so the fuzzy inference engine of a fuzzy logic system crafted by experts can be used as a prediction and optimization tool due to the engine tuning with computational intelligence [22,23]. Many research works have succeeded in employing fuzzy logic control systems (FLCS) in different industrial applications. FLCS is used in controlling processes that induce difficult mathematical modeling with high accuracy [24].

Kavka et al. structured and designed fuzzy logic controller for evaluating a simulated temperature control environment, showing that it is responsive to changes in the controlled process [25]. A FLCS was used to predict the surface hardness of the coating layer from titanium nitride—developed by physical vapor deposition—on aluminum alloy AL7075-T6 with respect to changes in DC power and nitrogen flow rate. The results of the fuzzy model demonstrated settlement between the fuzzy system and experimental work [26]. Another fuzzy logic model was designed to forecast the surface roughness of the same coated surface of the alloy, and the results showed an agreement between the fuzzy system and experimental results with 95.349% accuracy [27].

Several research works focused on using different fuzzy logic systems to control, predict, or optimize laser cladding parameters [28,29,30]. However, to the knowledge of the authors, no previous research focused on developing a FLCS to study the effect of the substrate surface roughness on the geometry and hardness of laser-cladded MG. Also, several studies have been conducted to examine the link between hardness and surface roughness. Grieve et al. [31], Wang and Feng [32], and Fischer and Elrod [33] explored surface roughness and its relationship with hardness. In addition, Sundarain and Lanibeil [34], Hasegawa et al. [35], and Miller et al. [36] investigated the relationship and discovered that hardness is inversely related to roughness.

In the present work, a fuzzy model was designed and implemented to predict and optimize the geometry and hardness of laser-cladded Fe-based MG single track on stainless steel substrate prepared at different surface roughness. The motivation behind proposing this model is that FLCS are inexpensive to design, cover a broad variety of operating conditions, and are easily adaptable in terms of natural language terminology. Such characteristics make the whole process time- and cost-effective. However, FLC has two significant shortcomings: it is unable to handle ambiguous data and comprehend human thought. Both of these issues are interconnected. If the data in the system is ambiguous, a person cannot deduce knowledge or relationships. In this study, authors inserted precise data into the proposed model to avoid ambiguity in data input, handling, and processing. This research work can help select the appropriate preparation technique for the substrate surface for maximum clad geometry that will lead to minimization of the number of cladding tracks in case of full surface cladding.

## 2. Laser Cladding of Fe-Based MG

**Figure 1:**
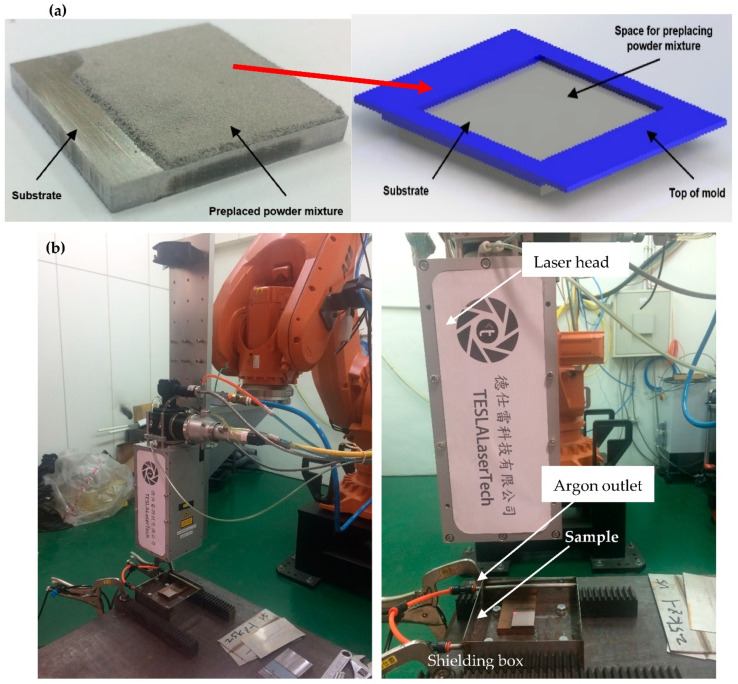
(**a**) Setup used to preplace the Fe-based amorphous powder, (**b**) laser cladding setup used.

F2229 SS was used as a substrate material. The chemical constituents of the substrate material are presented in Table 1. Four samples of substrate material were cut to a size of 30 × 30 × 3 mm thickness each. The surfaces of these four substrate samples were prepared following two different methods, namely by using SiC sandpaper (SP) (150-grit and 240-grit) and Al_2_O_3_ sandblasting (SB) (40-grit and 100-grit), to investigate effects of different surface roughness on the geometry and hardness of laser-cladded Fe-based metallic glass single track layer. The coating material used is Fe-based amorphous powder, placed as a 300 μm-thick layer on the prepared substrate samples with different surface roughness (Figure 1a). Next, the preplaced layer was laser-cladded under a continuous flow of Argon, to protect the molten pool from further oxidation, using a high-power diode laser machine (4.4 kW, wavelength 978–1025 ± 10 nm), Figure 1b. The laser power, scanning speed, and spot size were set to 2000 W, 45 mm/s, and 4 × 4 mm^2^, respectively, as described previously in our work [17].

**Figure 2:**
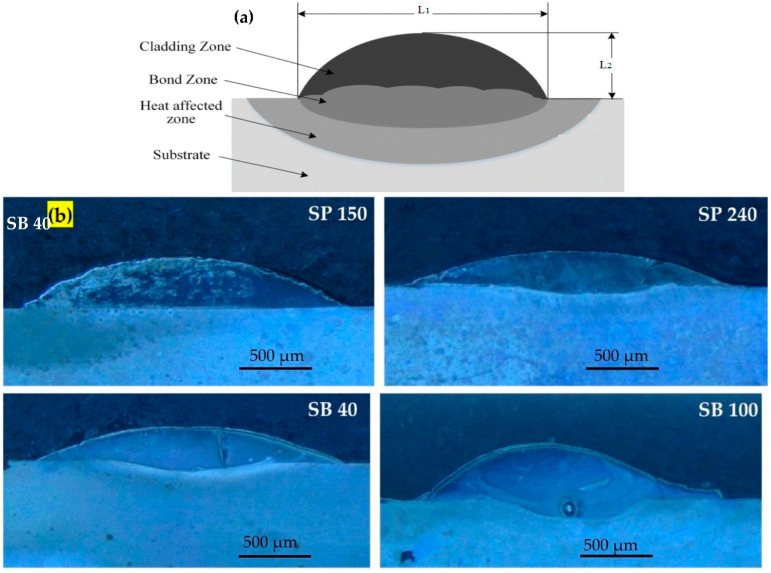
(**a**) Schematic of the cross-section of the laser-cladded single track with track height and width, (**b**) laser-cladded samples, with each sample presenting different surface roughness.

**Figure 3:**
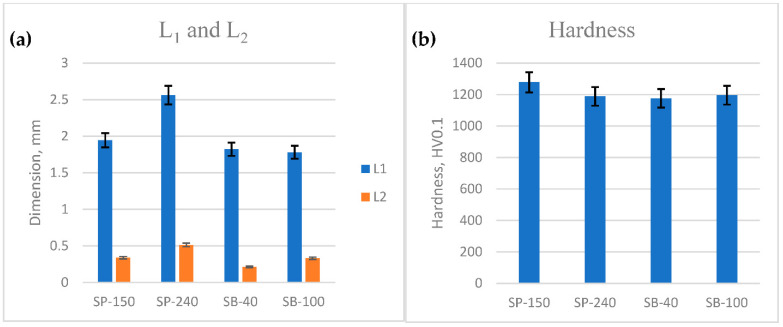
Experimental results of (**a**) L_1_ and L_2_, (**b**) hardness of laser-cladded single track.

Figure 2a shows a schematic for the cross-section of laser-cladded single track illustrating the relevant dimensions, the height of the track (L_1_), and the track width (L_2_). Figure 2b shows the laser-cladded samples, each original sample presenting different surface roughness. As it can be seen in Figure 2, the surface roughness of the substrate obtained by SP or SB processes has affected the geometry and the hardness of the single-cladded tracks of Fe-based MG. The results are presented in Table 2 and illustrated graphically in Figure 3.

Due to the superior cohesion force over the adhesion force between the molten coating material and the substrate, the surface roughness of the substrate was found to decrease. This led to an increment in the width and height of the cladded track. Conversely, the amorphous content percentage increased with increasing substrate surface roughness. Hardness measurements showed that the polished substrate exhibited higher hardness values than the sandblasted samples, which were affected by the amorphous content and the phases found within the coating layer. In conclusion, the polished substrate yielded optimum results in terms of geometry and hardness [37,38,39].

## 3. Fuzzy Logic Controller (FLC)

**Figure 4:**
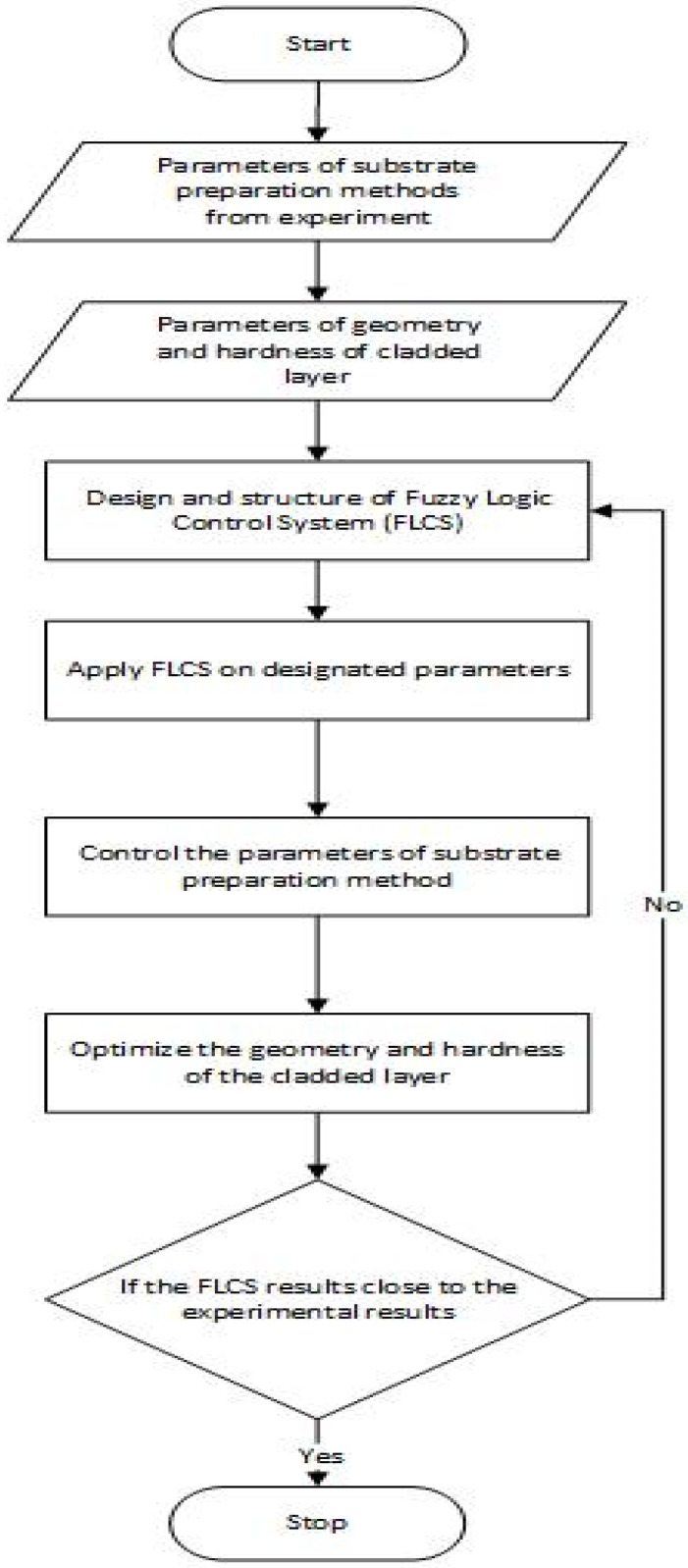
Flow chart of FLCS developed to optimize the geometry and hardness of laser-cladded Fe-based MG.

A fuzzy logic control system (FLCS) was used to predict which substrate surface preparation technique would optimize the geometry and hardness of the developed Fe-based MG single tracks. The flow chart of the FLCS used is shown in Figure 4.

The fuzzy logic control system analyzes analog input values in terms of discrete values of either 1 (true) or 0 (false). Fuzzy sets classify objects smoothly depending on membership, making them useful for approximation models [8]. A fuzzy logic system depends on the principle of assigning output based on the probability of the state of the input. Hereafter, If-Then rules are used, as they are the most appropriate to be utilized in the design of FLC [40].

### 3.1. Architecture of Fuzzy Logic Controller

**Figure 5:**
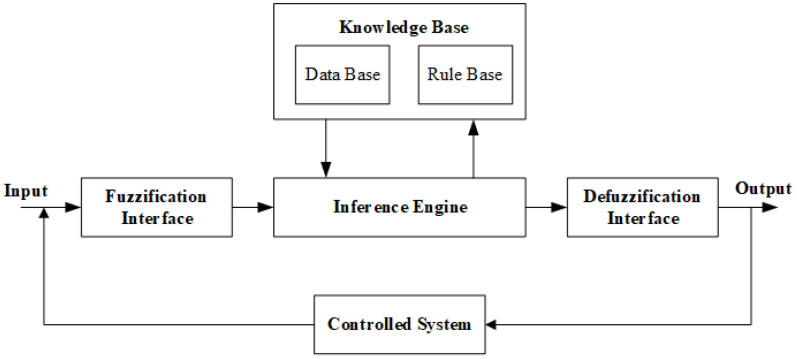
Structure of Fuzzy Logic Controller.

Fuzzifier, knowledge base, fuzzy rule base, and defuzzifier are the main components in the structure of a fuzzy controller for any controlled system, as shown in Figure 5.

The role of a fuzzifier in the fuzzy controller is to convert crisp input values into fuzzy values. Fuzzy knowledge base stores the knowledge about the input and output fuzzy relationships in the form of a membership function for each of them [41]. The fuzzy rule base uses the If-Then rule for joining membership functions of inputs and outputs. The inference engine is the core of any FLCS, as it performs approximate reasoning [42]. The defuzzification step represents the final stage in the fuzzy controller and is performed through the defuzzifier to convert the fuzzy values received from the fuzzy inference engine into new values [13]. The fuzzy logic toolbox of MATLAB is used to design and implement FLCS.

### 3.2. Inputs and Output Fuzzy System Variables

**Figure 6:**
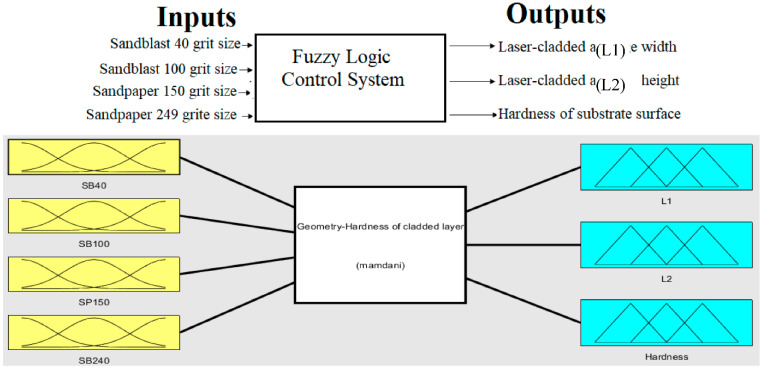
Geometry-hardness of laser cladded layer Fuzzy Logic Control System.

According to the results obtained from the experimental work, a four-input–three-output fuzzy logic control system was designed and applied to optimal values of geometry and hardness of laser-cladded Fe-based MG layer on a nickel-free high-nitrogen stainless-steel sample. The structure of the fuzzy system is shown in Figure 6.

Grit-size of the sandblast and sandpaper were used as inputs to the fuzzy system to optimize the geometry and hardness (outputs) of laser-cladded Fe-based MG layer.

### 3.3. Inputs and Outputs Membership Function

**Figure 7:**
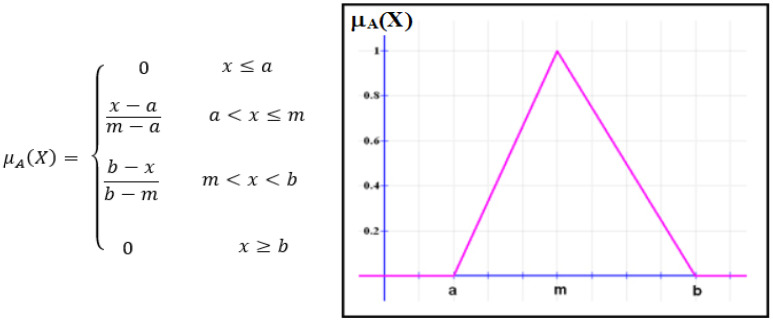
Fuzzy system membership function.

Fuzzy logic is used to characterize fuzziness. The membership function represents the best way to describe this fuzziness, as it expresses the degree of truth. For a fuzzy set (A), (X) can be expressed as µA:X → [0, 1], meaning the values of (X) are constrained within 0 and 1. The universe of discourse and degree of membership appear in the (x) axis and (y), respectively. The letters a and b represent the lower and upper limits of the triangular membership function, as shown in Figure 7 [43].

**Figure 8:**
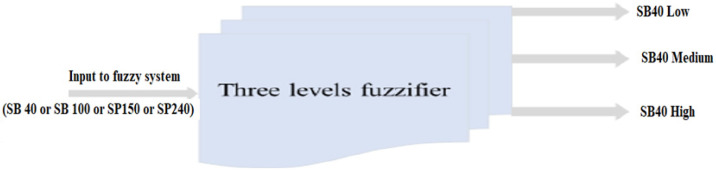
Fuzzification of fuzzy system inputs.

**Figure 9:**
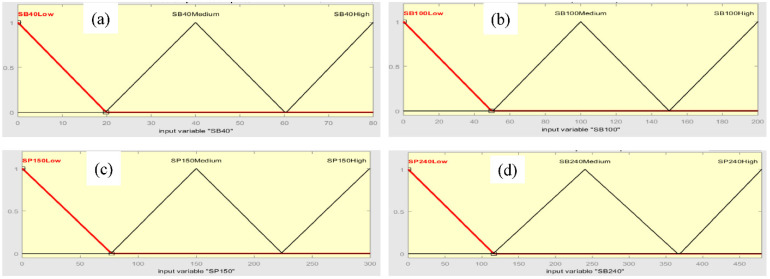
Triangular membership functions for fuzzy system inputs (**a**) SB 40, (**b**) SB 100, (**c**) SP 150, and (**d**) SP 240.

Fuzzification in fuzzy is performed by converting a new quantity of inputs into a fuzzy quantity by identifying the deterministic quantities as completely nondeterministic. The triangular membership function used to fuzzify each input to the designed fuzzy system into three fuzzy values (low, medium, and high) are shown in Figure 8. Triangular membership functions for the fuzzy system inputs (SB40, SB100, SP150, SP240) are shown in Figure 9a–d, respectively. Table 3 explains the range of fuzzy system inputs (sandblast and sandpaper) with three levels: low, medium, and high.

**Figure 10:**
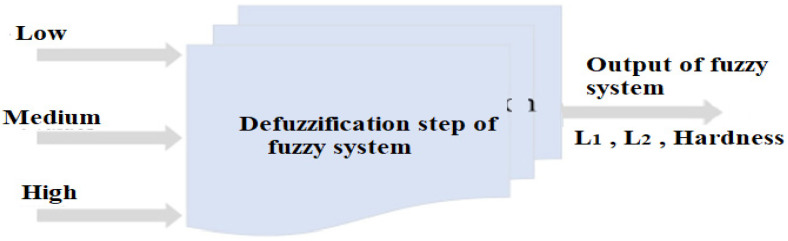
Defuzzification process in fuzzy system.

Defuzzification—required in FLCS—maps a fuzzy set to a new one through a number of rules that transform several variables into a fuzzy result for given fuzzy sets and corresponding membership degrees, as shown in Figure 10.

**Figure 11:**
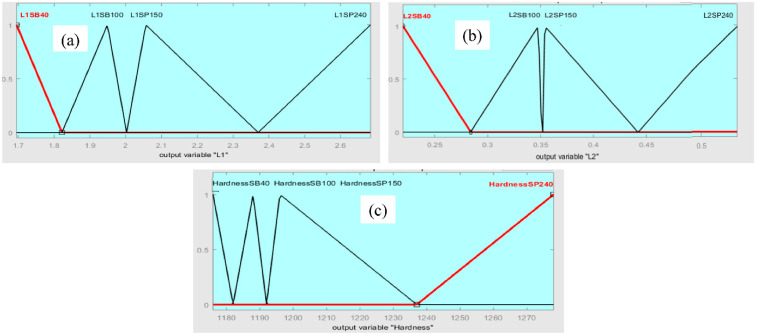
Outputs of fuzzy system.(**a**) geometry L1; (**b**) geometry L2; (**c**) hardness.

The triangular membership function is used in the defuzzification process for the fuzzy outputs with low, medium, and high levels to get geometry L_1_ and L_2_ (Figure 11a,b, respectively) and hardness (Figure 11c) of Fe-based MG, corresponding to the substrate surface preparation method (sandblast and sandpaper polishing). Levels of fuzzy system output are presented in Table 4.

### 3.4. FLC Base Rules

**Figure 12:**
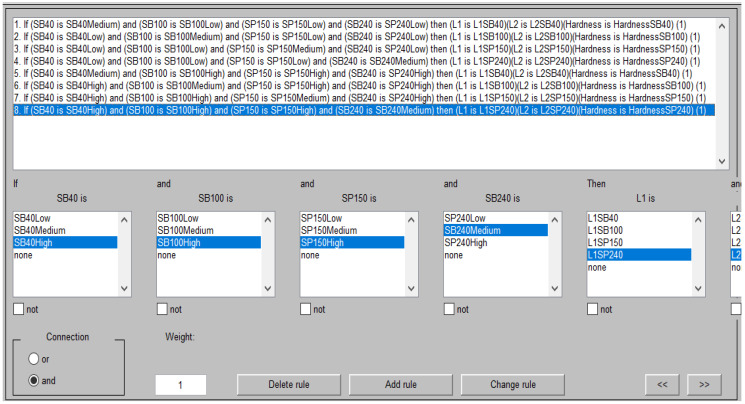
If-Then rules of geometry-hardness cladded layer fuzzy logic control system.

A fuzzy system uses rules based on the If-Then rule for assigning relationships between fuzzy system inputs and outputs. The rules that are used in the geometry-hardness cladded layer fuzzy logic control system are shown in Figure 12.

The results of the If-Then rules for the geometry-hardness fuzzy system yielded the maximum and minimum values of cladded layer geometry and hardness related to the sandblasting and sandpaper grit size used to prepare the substrate surface. The predicted values of geometry and hardness, constituting the main target of the fuzzy logic control system, are listed in Table 5.

A comparison was performed between experimental data [17] and the results of the geometry-hardness fuzzy system (Table 6) to verify the effectiveness of the designed FLCS in predicting the geometry and hardness of laser-cladded Fe-based MG single tracks. The values of the fuzzy logic control system are located between the experimental data values. The fuzzy logic control system yielded the best results for SP 240 (sandpaper grit size 240), thus agreeing with experimental data.

## 4. Conclusions

In this research, Fe-based MG was laser-cladded. The substrate was prepared using SiC sandpaper polishing (SP150 and SP240) and sandblast (SB40 and SB100) with fixed laser power, scanning speed, and spot size. Experimental results showed that the substrate surface roughness affected both geometry and hardness of the laser-cladded Fe-based MG single track. The geometry and hardness of the laser-cladded Fe-based MG single track were successively optimized according to the substrate surface roughness using a fuzzy logic control system (FLCS).

The substrate surface roughness was set as the input to the FLCS, and the geometry and hardness of laser-cladded Fe-based MG single tracks were set as the outputs and optimized. The results of built FLCS were compared with the experimental results. Fuzzy logic control system results exhibited agreement with experimental results and showed that the best results were afforded by SP240,—this was also demonstrated experimentally.

The results obtained from FLCS, which is considered a type of artificial intelligence, demonstrated that a fuzzy logic control system is an easy and inexpensive technology that can be used in prediction and optimization of laser cladding of MGs. The obtained results illustrate the efficacy and adequacy of the FLCS proposed.

The work may be further extended to examine the strength and degree of thermal insulation in the cladded parts for structural applications.

## Figures and Tables

**Table 1:** Chemical constituents of the substrate material.

Material	S	P	C	Ni	Cu	Si	N	Mn	Cr	Mo	Fe
F2229 SS	0.01	0.03	0.08	0.1	0.3	0.7	>0.90	1.5	19	21	Balance

**Table 2:** Experimental results.

Sample	L_1_, mm	L_2_, mm	Hardness, HV0.1
SP-150 (Sand Paper with 150 grit)	1.946 ± 0.110	0.338 ± 0.016	1278
SP-240 (Sand Paper with 200 grit)	2.562 ± 0.122	0.514 ± 0.015	1188
SB-40 (Sand Blasting with 40 grit)	1.822 ± 0.127	0.212 ± 0.008	1176
SB-100 (Sand Blasting with 100 grit)	1.780 ± 0.089	0.330 ± 0.018	1196

**Table 3:** Membership Functions of Fuzzy System Inputs.

Fuzzy System Inputs Variables	Membership Function Used	Range of Inputs		
		Low	Medium	High
Sandblast (SB 40)	Triangular MF	0–20	20–60	60–80
Sandblast (SB 100)	Triangular MF	0–45	45–150	150–200
Sandpaper (SP 150)	Triangular MF	0–75	75–225	225–300
Sandpaper (SP 240)	Triangular MF	0–125	125–375	375–475

**Table 4:** Membership functions of fuzzy system outputs.

Fuzzy System Outputs Variables	Membership Function Used	Range of Outputs			
		SB 40	SB 100	SP 150	SP 240
L_1_, mm	Triangular MF	1.700–1.835	1.845–2.000	2.010–2.385	2.386–2.680
L_2_, mm	Triangular MF	0.000–0.289	0.290–0.350	0.351–0.440	0.450–0.610
Hardness, HV0.1	Triangular MF	0.000–1182.0	1182.1–1192.0	1192.2–1238.0	1238.3–1300.0

**Table 5:** Output results of fuzzy system.

**Values**	**Inputs**				**Outputs**		
	**SB 40**	**SB100**	**SP 150**	**SP 240**	**L_1_, mm**	**L_2_, mm**	**Hardness, HV0.1**
	40	0	0	0	1.73	0.240	1180
	0	100	0	0	1.92	0.328	1190
	0	0	150	0	2.05	0.383	1210
	0	0	0	240	2.11	0.501	1260

**Table 6:** Values of geometry and hardness from the fuzzy system and experimental data.

Parameters	L_1_, mm		L_2_, mm		Hardness, HV0.1	
	Experimental Data	Fuzzy Result	Experimental Data	Fuzzy Result	Experimental Data	Fuzzy Result
SB 40	1.822 ± 0.127	1.73	0.212 ± 0.008	0.240	1176	1180
SB 100	1.780 ± 0.089	1.92	0.330 ± 0.018	0.328	1196	1190
SP 150	1.946 ± 0.110	2.05	0.338 ± 0.016	0.383	1278	1210
SP 240	2.562 ± 0.122	2.11	0.514 ± 0.015	0.501	1188	1260

## Data Availability

All the data are clearly available in the manuscript.

## References

[1] Siddiqui A.A., Dubey A.K. (2021). Recent trends in laser cladding and surface alloying. Opt. Laser Technol..

[2] Bourahima F., Helbert A.L., Rege M., Ji V., Solas D., Baudin T. (2019). Laser cladding of Ni based powder on a Cu-Ni-Al glassmold: Influence of the process parameters on bonding quality and coating geometry. J. Alloys Compd..

[3] Zeng C., Tian W., Liao W.H., Hua L., He W., Hua L. (2016). Microstructure and porosity evaluation in laser-cladding deposited Ni-based coatings. Surf. Coat. Technol..

[4] Comesaña R., Quintero F., Lusquiños F., Pascual M.J., Boutinguiza M., Durán A., Pou J. (2010). Laser cladding of bioactive glass coatings. Acta Biomater..

[5] Penide J., Lusqui F., Quintero F., Riveiro A., Boutinguiza M., Pou J., Arias-González F., del Val J., Comesaña R., Penide J. (2016). Fiber laser cladding of nickel-based alloy on cast iron. Appl. Surf. Sci..

[6] Li Y. Laser Cladding of Alumina Material Coating: Effects on Deposition Quality. Proceedings of the ASME 2016 11th International Manufacturing Science and Engineering Conference.

[7] Gao Y.-L., Shen J., Sun J.-F., Wang G., Xing D.-W., Xian H.-Z., Zhou B.-D. (2003). Crystallization behavior of ZrAlNiCu bulk metallic glass with wide supercooled liquid region. Mater. Lett..

[8] Suryanarayana C., Inoue A. (2013). Iron-based bulk metallic glasses. Int. Mater. Rev..

[9] Maddala D.R., Mubarok A., Hebert R.J. (2010). Sliding wear behavior of Cu50Hf41.5Al8.5 bulk metallic glass. Wear.

[10] Williams E., Lavery N. (2017). Laser processing of bulk metallic glass: A review. J. Mater. Process. Technol..

[11] Yanfang W., Qinglong L., Lijun X., Zhiqiang S. (2014). Laser Cladding Fe-Cr-Si-P Amorphous Coatings on 304L Stainless. Rare Met. Mater. Eng..

[12] Zhang W., Tao P., Tu Q., Li D., Yang Y. (2017). Effect of laser surface melting on bulk metallic glass: Investigation of microstructure, microhardness, friction and wear properties. J. Alloys Compd..

[13] Ibrahim M.Z., Sarhan A.D., Shaikh M.O., Kuo T.Y., Yusuf F., Hamdi M. (2019). Investigate the effects of the laser cladding parameters on the microstructure, phases formation, mechanical and corrosion properties of metallic glasses coatings for biomedical implant application. Additive Manufacturing of Emerging Materials.

[14] Wu X.L., Hong Y.S. (2000). Microstructure of Zr-alloyed coating using pulsed laser. Surf. Coat. Technol..

[15] Li R., Jin Y., Li Z., Zhu Y., Wu M. (2014). Effect of the remelting scanning speed on the amorphous forming ability of Ni-based alloy using laser cladding plus a laser remelting process. Surf. Coat. Technol..

[16] Liu H., Qin X., Huang S., Hu Z., Ni M. (2018). Geometry modeling of single track cladding deposited by high power diode laser with rectangular beam spot. Opt. Lasers Eng..

[17] Ibrahim M.Z., Sarhan A.A.D., Kuo T.Y., Yusuf F., Hamdi M., Chien C.S. (2018). Investigate the effects of the substrate surface roughness on the geometry, phase transformation, and hardness of laser-cladded Fe-based metallic glass coating. Int. J. Adv. Manuf. Technol..

[18] Singh N., Hameed P., Ummethala R., Manivasagam G., Prashanth K.G., Eckert J. (2020). Selective laser manufacturing of Ti-based alloys and composites: Impact of process parameters, application trends, and future prospects. Mater. Today Adv..

[19] Shamsaei N., Yadollahi A., Bian L., Thompson S.M. (2015). An overview of Direct Laser Deposition for additive manufacturing; Part II: Mechanical behavior, process parameter optimization and control. Addit. Manuf..

[20] Tamanna N., Crouch R., Naher S. (2019). Progress in numerical simulation of the laser cladding process. Opt. Lasers Eng..

[21] Bakhtiyari A.N., Wang Z., Wang L., Zheng H. (2021). A review on applications of artificial intelligence in modeling and optimization of laser beam machining. Opt. Laser Technol..

[22] Klement E.P., Slany W. Fuzzy Logic in Artifcial Intelligence, Vienna. Proceedings of the 8th Austrian Artificial Intelligence Conference, FLAI’93.

[23] Yager R.R. (1997). Fuzzy logics and artificial intelligence. Fuzzy Sets Syst..

[24] Precup R.E., Hellendoorn H. (2011). A survey on industrial applications of fuzzy control. Comput. Ind..

[25] Kavka C., Roggero P., Apolloni J. An Architecture for Fuzzy Logic Controllers Evolution and Learning in Microcontroller based Environments. Proceedings of the IX Congreso Argentino de Ciencias de la Computación.

[26] Zalnezhad E., Sarhan A.A.D., Hamdi M. (2013). A fuzzy logic based model to predict surface hardness of thin film TiN coating on aerospace AL7075-T6 alloy. Int. J. Adv. Manuf. Technol..

[27] Zalnezhad E., Sarhan A.A.D. (2014). A fuzzy logic predictive model for better surface roughness of Ti-TiN coating on AL7075-T6 alloy for longer fretting fatigue life. Meas. J. Int. Meas. Confed..

[28] Zeinali M., Khajepour A. (2010). Development of an adaptive fuzzy logic-based inverse dynamic model for laser cladding process. Eng. Appl. Artif. Intell..

[29] Sohrabpoor H. (2017). Perspective of Applying Adaptive Neuro Fuzzy Inference System (ANFIS) in Laser Cladding of Graphene-Metal Alloys Predictive quality modeling of polymer and metal parts fabricated by SLS and SLM additive manufacturing processes View project. J. Nanotechnol..

[30] Nair A., Ramji V., Durai Raj R., Veeramani R. (2020). Laser cladding of Stellite 6 on EN8 steel—A fuzzy modelling approach. Mater. Today Proc..

[31] Grieve D.J., Kaliszer H., Rowe G.W. The effects of cutting conditions on bearing area parameters. Proceedings of the 9th International Machine Tool Design and Research Conference.

[32] Wang X., Feng C.X. (2002). Development of Empirical Models for Surface Roughness Prediction in Finish Turning. Int. J. Adv. Manuf. Technol..

[33] Fischer H.L., Elrod J.T. (1971). Surface finish as a function of tool geometry and feed—A theoretical approach. Microtecnic.

[34] Sundarain R.M., Lanibeil B.K. (1981). Mathematical models to predict surface finish in fine turning of steel, Pans I and II. Int. J. Prod. Res..

[35] Hascgawa H., Seireg A., Lindberg R.A. (1976). Surface roughness model for turning. Tribology.

[36] Miller J.C., de Vor R.E., Southerland J.W. Surface roughness characteristics for turning 380 and 390 aluminum casting alloys. Proceedings of the North American Manufacturing Research Conference.

[37] Daroonparvar M., Bakhsheshi-Rad H.R., Saberi A., Razzaghi M., Kasar A.K., Ramakrishna S., Menezes P.L., Misra M., Ismail A.F., Sharif S. (2022). Surface modification of magnesium alloys using thermal and solid-state cold spray processes: Challenges and latest progresses. J. Magnes. Alloy..

[38] John M., Kuruveri U.B., Menezes P.L. (2022). Laser Cladding-Based Surface Modification of Carbon Steel and High-Alloy Steel for Extreme Condition Applications. Coatings.

[39] Qu M., Liang T., Hou J., Liu Z., Yang E., Liu X. (2022). Laboratory study and field application of amphiphilic molybdenum disulfide nanosheets for enhanced oil recovery. J. Pet. Sci. Eng..

[40] Liu H., Coghill G.M., Barnes D.P. (2009). Fuzzy qualitative trigonometry. Int. J. Approx. Reason..

[41] Patyra M.J., Grantner J.L. (1999). Hardware implementations of digital fuzzy logic controllers. Inf. Sci..

[42] Adilova N.E. (2020). Consistency of fuzzy if-then rules for control system. Advances in Intelligent Systems and Computing.

[43] Pedrycz W. (1994). Why triangular membership functions?. Fuzzy Sets Syst..

